# Do We Know the Long‐Term Effects of the Most Popular Traditional Swallow Maneuvers on the Submental Muscles?

**DOI:** 10.1111/joor.13862

**Published:** 2024-09-30

**Authors:** Ayşe Kübra Söyler, Nefati Kıylıoğlu, Selen Serel Arslan, Numan Demir, Mustafa Gök, Ersen Ertekin, Tülin Düger

**Affiliations:** ^1^ Faculty of Health Science, Physiotherapy and Rehabilitation Aydın Adnan Menderes University Aydın Turkey; ^2^ Department of Neurology, Faculty of Medicine Aydın Adnan Menderes University Aydın Turkey; ^3^ Faculty of Physical Therapy and Rehabilitation Hacettepe University Ankara Turkey; ^4^ Department of Radiology, Faculty of Medicine Aydın Adnan Menderes University Aydın Turkey; ^5^ Department of Health Sciences, Faculty of Medicine and Health University of Sydney Sydney Australia; ^6^ Department of Radiology, Faculty of Medicine Hitit University Corum Turkey

**Keywords:** deglutition, effortful swallow, electromyography, Masako maneuver, muscle strength, muscle thickness

## Abstract

**Background:**

Various trainings focus on the submental muscles (SMs) for dysphagia rehabilitation because of their importance for swallowing safety and efficiency. According to the current literature, swallow‐specific tasks may be optimal exercises for dysphagia. The effortful swallow (ES) and the Masako maneuver (MM) are the most commonly used swallow‐specific tasks in the clinical settings for dysphagia for years, but long‐term effects for these trainings is insufficient.

**Objectives:**

This study aims to investigate and compare the effects of ES and MM on SM activity, strength and thickness.

**Methods:**

Thirty‐seven healthy adults were randomised to ES, MM and control groups, and ES and MM groups completed 6 weeks of swallowing training. Participants in both training groups performed a total of 120 swallows in each session, while control group did not participate in any swallowing training. Surface electromyography was used to evaluate SM activity, digital dynamometer for SM strength and ultrasonography for SM thickness.

**Results:**

Both trainings did not change SM activity (*p* > 0.05), but increased SM strength (*p* < 0.05). MM increased the thickness of all SM (*p* < 0.05), and ES increased the thickness of mylohyoid (right, left) and digastric muscle (right) (*p* < 0.05), and there was no change in all evaluation parameters in the control group (*p* > 0.05). Also, trainings were not superior to each other in any parameter (*p* > 0.05).

**Conclusion:**

The results of this study provided new evidence to the literature to show that ES and MM trainings are effective for improving SM strength and thickness. Considering that SM is important in terms of swallowing safety and effectiveness, it is thought that both trainings may be promising by increasing the strength and mass of SM, especially in individuals with reduced SM strength and mass.

## Graphical abstract

Due to the role of submental muscles in swallowing safety and efficiency, the results of this study suggest that both ES and MM may be promising, especially by increasing the strength and mass of the submental muscles.

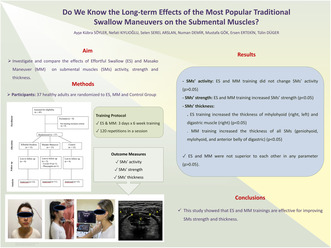

## 1 Background

Dysphagia is a serious condition that causes important health problems such as malnutrition, dehydration, aspiration pneumonia, in addition to all these physical problems, it leads to social isolation of patients and their caregivers and decrease their quality of life, and psychologically affects them negatively. Therefore, timely and adequate care and rehabilitation of dysphagia is critical. Interventions for dysphagia often comprise compensatory and rehabilitative techniques and exercise training is one of the most commonly used rehabilitation technique in the clinical settings [1]. The primary goal of exercise training in dysphagia is to increase the strength and endurance of the muscles involved in swallowing function, hence promoting swallowing safety and efficiency [2].

Due to the importance of the submental muscles (SMs) in swallowing function, several exercises used in dysphagia rehabilitation focus on improving them. As it is known, the SMs play a primary role in controlling the movement of the hyoid bone during oral motor functions (swallowing, chewing, phonation and breathing) [3, 4] and its most significant functions is the movement of the hyolaryngeal complex. Reduced hyolaryngeal displacement, a key event in swallowing, results in insufficient upper oesophageal sphincter opening, may result in aspiration and pharyngeal residue [3, 4]. Furthermore, the mylohyoid, the geniohyoid and the anterior belly of the digastric muscle, participate not only in hyoid elevation but also in jaw opening [4, 5, 6]. Despite being less widely known, new research has discovered two additional significant functions of the SM including participation in bolus formation during chewing and contributing to the tongue pressure against the hard palate by stimulating the floor of the mouth and elevating the tongue [3, 6]. Due to these important tasks, structural and functional improvement of SM has an important place in improving swallowing safety and efficiency. At this point, the selection of appropriate exercise training gain importance in order to achieve maximum outcomes. In terms of clinical outcomes, choosing a purposeful exercise that meets the training principles is essential in terms of maximise clinical outcomes [7]. Task specificity is one of the most essential considerations in this regard [7, 8]. The current view on this issue is that swallow‐specific tasks may be optimal exercises for dysphagia. In this context, effortful swallow (ES) and Masako maneuver (MM/tongue‐hold swallow) are the most frequently used swallow‐specific tasks for dysphagia rehabilitation for years.

Despite its widespread clinical popularity, it is seen that studies on the training effects of ES and MM in both healthy individuals and individuals with dysphagia are insufficient and most of the existing studies focus on the short‐term physiological effects [8, 9, 10, 11]. According to these studies, ES increases oral and pharyngeal pressure and duration, hyolaryngeal movement and SM activity, and the MM enhances SM activity, posterior pharygeal wall movement, pharyngeal pressure and intraoral pressure [8, 9, 10, 12, 13, 14, 15, 16]. In addition, a recent study on the swallow‐specific task reported that ES was superior to other swallow maneuvers, especially in terms of selective SM contraction and authors suggested that for future studies investigate whether this trainings increase SM strength or thickness [8]. In the light of these studies, ES and MM trainings appear to be promising in terms of SM strength and tickness. However, studies on this issue are very limited. Only a few studies [17, 18, 19] investigated the effect of ES on SM activity in the context of long‐term training and no studies on the effect of either trainings on SM thickness were conducted to date.

The lack of study on the training effects of these maneuvers, which have been used often in clinical settings for many years, highlights the need for more research on this topic in order to establish the best evidence‐based clinical practice. In addition, it will be clinically valuable for exercise selection to determine which of these two similar exercises is superior in terms of improving the SM. First of all, determining the effects of ES and MM training on SM in healthy individuals will be valuable in terms of forming a basis for future studies in elderly and individuals with dysphagia. For these reasons, the current study aims to investigate and compare the effects of ES and MM on SM activity, strength and thickness in healthy adults.

## 2 Materials and Methods

### 2.1 Study Design and Participants

This study was conducted in cooperation with Hacettepe University Faculty of Physical Therapy and Rehabilitation, Adnan Menderes University, Faculty of Medicine and Adnan Menderes University, Faculty of Health Sciences, Department of Physiotherapy and Rehabilitation. The study protocol was approved by Adnan Menderes University, Faculty of Health Sciences, Non‐Invasive Clinical Research Ethics Committee (Ethical Approval Number E‐15189967‐050.02.04‐145 385). Volunteers for the study were informed about the study and signed an informed consent form.

Healthy individuals between the ages of 18 and 28 years were included in the study. Individuals with disc herniation, mechanical neck pain or any pathology in the cervical region, temporomandibular joint problems that may affect joint biomechanics and muscle functions, any neurological or systemic disease or those undergoing head and neck surgery or radiotherapy were excluded from the study [20].

Thirty‐seven healthy individuals meeting the inclusion criteria were allocated to effortful swallow (*n* = 12), Masako maneuver (*n* = 13) or control group (*n* = 12) by computer‐based randomisation. Due to health problems including COVID‐19 and pharyngitis during the exercise training, two volunteers in the MM group were unable to complete the training and therefore excluded from the study.

### 2.2 Outcome Measures

All outcome measures were assessed twice: once at the start of the training and once after the 6‐week training. The participants' demographic characteristics, including gender, age, height and weight were recorded.

### 2.3 Evaluation of SMs' Activity

Surface electromyography (sEMG) (Nihon Kohden Neuropack 2 MEB 7102K) was employed to objectively assess the neuromuscular activity of the SM during normal and effortful swallow task. This evaluation was performed at electromyography laboratory by an experienced neurologist who is blind to group allocation.

After cleaning the skin with an alcohol wipe, silver–silver chloride disc electrodes (0.9 cm) were placed on the submental region 1 cm to the right and left sides of the chin. To prevent artefacts during sEMG data collection, cables and electrodes were fixed with adhesive tape. sEMG signals were filtered (50–500 Hz), averaged. Amplitude scale was set as 100–400 mV, sweep time was 10 s and delay was set as 3 s. All sEMG were recorded during normal swallow and effortful swallow tasks, respectively. Each participant received one trial and three successful tasks were recorded. For the normal swallow task, the participant was asked to ‘swallow your saliva comfortably’ as in daily life. For effortful swallow, the participant was asked to ‘Squeeze hard with all your muscles as you swallow your saliva’. Mean sEMG amplitudes were recorded as microvolt for data analysis [8].

### 2.4 Evaluation of SMs' Thickness

Ultrasonography (Samsung Medison Co. Ltd., Seoul, Korea) was used to assess the thickness of the SM (geniohyoid, mylohyoid and anterior belly of digastric). All muscle thickness measurements were performed by the an experienced radiologist who is blinded to groups.

**FIGURE 1:**
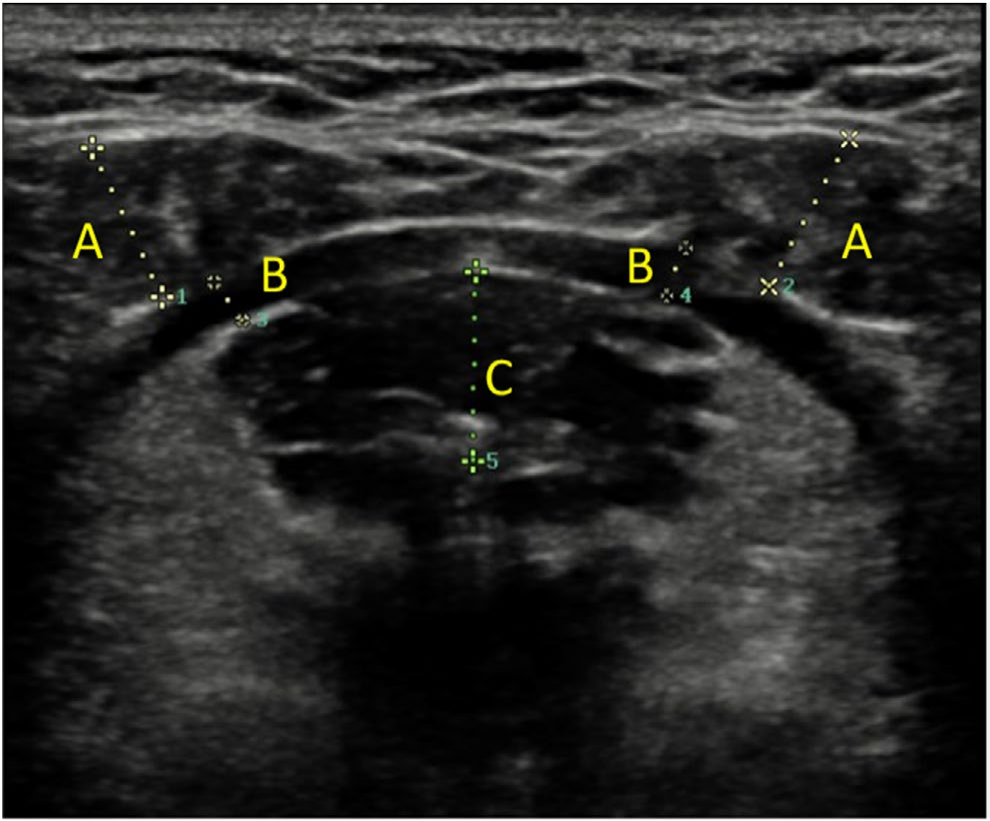
Submental muscles thickness measurement: (A) anterior belly of digastric muscles, (B) mylohyoid muscles and (C) geniohyoid muscle.

For measurement, an L3‐12A wideband, high‐resolution linear probe with a frequency range of 3–12 mHz was used. During the assessment, the participant sat erect in a chair with a neutral head posture [21]. The probe was placed between the hyoid bone and the chin with minimal pressure to avoid compression of soft tissues and musculature. The accuracy of the probe placement was confirmed by the symmetrical images of the right and left anterior belly of digastric muscle on the ultrasonography system screen [21]. The midpoint of the muscle was used as a reference point for thickness measurement and the distance between the upper and lower borders of muscle was measured. The digastric and mylohyoid muscles were measured bilaterally (Figure 1). All measurements were performed three times for each muscle and the mean value in millimetres was recorded for analysis [22].

### 2.5 Evaluation of SMs' Strength

Jaw opening force was measured with Lafayette Hand‐held digital dynamometer (Model 01165 Lafayette Instrument Inc., USA) to evaluate SM strength [23]. For evaluation, the participant sat on a chair without back support placed in front of the wall and leaned his back and head against the wall. Thus, compensatory movements were prevented during the evaluation. The head of the digital dynamometer was placed under the participant's chin and the individual was instructed to open their jaws as forcefully as possible for 5 s. After one trial, three measurements were taken with a 60 s rest interval between them and the average value was recorded for analysis [23, 24, 25].

### 2.6 Interventions

First, each participant underwent a face‐to‐face interview, received instructions on the exercise training protocol and practiced the exercise training prior to beginning. Thus, it was ensured that the participant correctly performed the appropriate exercise training. Control group continued their daily routine did not participate in any swallowing training. Volunteers in both training groups participated exercise training 3 days per week, not on consecutive days, for 6 weeks. Due to the global pandemic, exercise training was undertaken online in groups of two to three persons under the supervision a physical therapist with 5 years of experience in dysphagia rehabilitation. The participants sat on a chair along the exercise training. For ES training, participants were instructed by physical therapist to ‘Squeeze hard with all your muscles as you swallow your saliva’ [8] and MM training ‘Stick your tongue out of your mouth, hold it between your incisors and swallow your saliva’ every 10 s [8, 26]. Participants in both exercise training group performed a total of 120 swallow in a session [26, 27]. To prevent fatigue, a session was divided into two parts with 2 min of rest between them. Thus, a session was completed in an average of 22 min. Between swallowing exercises, participants were allowed to drink water as needed [21, 28].

### 2.7 Statistical Analysis

The SPSS software (version 26; SPSS Inc., Chicago, IL) was used for statistical analysis. Visual (histogram analysis and probability plots) and analytical (Shapiro–Wilk tests) methods were used to determine normality assumption. Depending on the normality of the data, paired samples *t*‐test or Wilcoxon signed‐rank test was used in the analysis of intra‐group changes. The one‐way analysis of variance test (post hoc: Tukey test) or the Kruskal–Wallis analysis of variance (post hoc: Mann–Whitney *U*‐test with Bonferroni correction) test were used for to investigate independent group comparisons for measurements and delta (difference values for pre and post test) values. *p* < 0.05 was accepted as a statistically significant result.

## 3 Results

In terms of age, height, body weight and gender, there was no significant difference among groups (*p* > 0.05) (Table 1). No significant difference was detected among groups in all variables (muscle activity, thickness and strength) regarding baseline values of the participants (*p* > 0.05) (Tables 2 and 3).

**TABLE 1:** Descriptive characteristics of participants.

	ES (*n* = 12) median (IQR)	MM (*n* = 11) median (IQR)	Control (*n* = 12) median (IQR)	*p*
Age (years)	20 (20–21)	20 (19–21)	20 (19–22)	0.882
Height (cm)	170 (168–178)	168 (165–175)	171 (162–178)	0.772
Body weight (kg)	61 (56–75)	72 (57–80)	68 (59–76)	0.601
**Gender**	***N* (%)**	***N* (%)**	***N* (%)**	
Women	8 (62)	8 (67)	8 (62)	
Men	5 (38)	4 (33)	5 (38)	

Abbreviations: cm, centimetre; ES, effortful swallow; kg, kilogram; MM, Masako maneuver.

**TABLE 2:** Changes in submental muscles activity and strength before and after training.

	ES (*n* = 12)	MM (*n* = 11)	Control (*n* = 12)	Inter‐group *p*
Suprahyoid muscle activity during normal swallow (mV)				
Pre‐training (IQR)	117 (82–175)	140 (110–207)	150 (96–220)	0.374 (kw = 1.965)
Post‐training (IQR)	107 (80–129)	117 (103–150)	110 (70–180)	0.509 (*F* = 0.689)
Intra‐group *p*	0.184 (*t* = 1.417)	0.173 (*t* = 1.466)	0.142 (*t* = 1.596)	
Suprahyoid muscle activity during effortful swallow (mV)				
Pre‐training (IQR)	171 (127–231)	173 (160–210)	250 (117–270)	0.621 (kw = 0.953)
Post‐training (IQR)	173 (107–245)	147 (123–250)	220 (107–300)	0.783 (*F* = 0.247)
Intra‐group *p*	0.912 (*t* = −0.113)	0.656 (*t* = 0.459)	0.912 (*t* = −0.113)	
Suprahyoid muscle strength (N)				
Pre‐training (IQR)	64 (58.4–67.7)	70 (59.2–75.3)	64 (56.3–73.1)	0.507 (*F* = 0.695)
Post‐training (IQR)	72 (62.3–76.4)	74 (70.3–81.7)	63 (52.1–74.2)	0.015* (*F* = 4.87)^a^
Intra‐group *p*	0.0001* (*t* = −7.152)	0.014* (*t* = −2.963)	0.281 (*t* = 1.148)	

Abbreviations: ES, effortful swallow; *F*, one‐way analysis of variance (post hoc: Tukey test); kw, Kruskal–Wallis variance analysis (post hoc: Mann–Whitney *U*‐test with Bonferroni correction); MM, Masako maneuver; mV, microvolt; *t*, paired samples *t* test; *z*, Wilcoxon signed‐rank test.

* *p* < 0.05 statistically significant difference, all descriptive statistics are expressed as median (25th–75th percentile).

^a^ Significant difference between MM and control group.

**TABLE 3:** Changes in submental muscles thickness before and after training.

	ES (*n* = 12)	MM (*n* = 11)	Control (*n* = 12)	Inter‐group *p*
Geniohyoid (mm)				
Pre‐training (IQR)	7.98 (7.53–11.26)	7.05 (6.46–8.73)	8.73 (7.2–9.56)	0.118 (kw = 4.277)
Post‐training (IQR)	8.98 (7.65–11.32)	8.33 (7.56–9.56)	8.80 (7.53–10.03)	0.572 (kw = 1.116)
Intra‐group *p*	0.089 (*t* = −1.866)	0.001* (*t* = −4.373)	0.132 (*t* = −1.641)	
Mylohyoid—right (mm)				
Pre‐training (IQR)	2.08 (1.61–3.48)	2.13 (1.76–2.66)	2.03 (1.86–2.23)	0.57 (*F* = 0.573)
Post‐training (IQR)	2.71 (1.96–4.10)	2.70 (2.33–3.20)	2.03 (1.66–2.46)	0.05* (*F* = 3.284)^a^
Intra‐group *p*	0.01* (*t* = −3.121)	0.042* (*t* = −2.33)	0.68 (*t* = −0.424)	
Mylohyoid—left (mm)				
Pre‐training (IQR)	2.22 (1.62–3.43)	2.06 (1.90–2.86)	2.10 (1.90–2.23)	0.983 (kw = 0.033)
Post‐training (IQR)	2.78 (2.16–3.95)	2.66 (2.43–2.96)	1.86 (1.73–2.40)	0.024* (kw = 7.494)^a^
Intra‐group *p*	0.003* (*t* = −3.75)	0.007* (*t* = −3.358)	0.188 (*t* = 1.414)	
Digastric—right (mm)				
Pre‐training (IQR)	6.53 (5.74–9)	6.76 (6.30–7.23)	7.53 (6.50–7.76)	0.803 (kw = 0.439)
Post‐training (IQR)	7.17 (5.89–9.16)	7.53 (6.96–8.40)	7.20 (6.73–8.13)	0.833 (*F* = 0.184)
Intra‐group *p*	0.017* (*t* = −2.805)	0.045* (*t* = −2.29)	0.679 (*t* = −0.426)	
Digastric—left (mm)				
Pre‐training (IQR)	6.60 (6.18–8.62)	6.60 (6.2–7.23)	7.41 (6.63–7.8)	0.648 (*F* = 0.44)
Post‐training (IQR)	7.17 (5.9–9.15)	7.26 (6.86–8.36)	7.30 (6.83–7.9)	0.418 (*F* = 0.898)
Intra‐group *p*	0.09 (*t* = −1.858)	0.013* (*z* = −2.497)	0.583 (*t* = 0.509)	

Abbreviations: ES, effortful swallow; *F*, one‐way analysis of variance (post hoc: Tukey test); kw, Kruskal–Wallis variance analysis (post hoc: Mann–Whitney *U* test with Bonferroni correction); MM, Masako maneuver; mV, microvolt; *t*, paired samples *t* test; *z*, Wilcoxon signed‐rank test.

* *p* < 0.05 statistically significant difference, all descriptive statistics are expressed as median (25th–75th percentile).

^a^ Significant difference between ES and control group.

After 6 weeks of training, there was no significant change in all groups in the mean sEMG amplitudes obtained during both normal swallow and effortful swallow tasks (*p* > 0.05) (Tables 2 and 4).

**TABLE 4:** Comparison of submental muscles activity, strength and thickness changes between groups (difference of pre‐post training).

Δ (pre–post)	ES (*n* = 12)	MM (*n* = 11)	Control (*n* = 12)	Inter‐group *p*
Suprahyoid muscle activity during Normal swallow (mV)	18.5 (−2.25 to 47)	30 (−10 to 87)	21 (−24 to 60)	0.876 (*F* = 0.132)
Suprahyoid muscle activity during effortful swallow (mV)	−2 (−62.25 to 61.25)	30 (−70 to 60)	7 (−80 to 43)	0.895 (*F* = 0.112)
Suprahyoid muscle strength (N)	−7.06 (−10.13 to −4.65)	−10.4 (−16.4 to 0.03)	0.49 (−1.11 to 2.43)	0.002* (*F* = 7.678)^a^, ^b^
Geniohyoid (mm)	−0.15 (−0.95 to 0.18)	−0.66 (−1.43 to −0.4)	−0.17 (−0.47 to 0.13)	0.092 (*F* = 2.583)
Mylohyoid—right (mm)	−0.67 (−1.07 to 0.11)	−0.46 (−0.67 to −0.2)	0 (−0.17 to 0.2)	0.071 (*F* = 2.887)
Mylohyoid—left (mm)	−0.7 (−0.99 to −0.15)	−0.53 (−0.8 to 0.03)	0.13 (−0.07 to 0.2)	0.002* (*F* = 8.036)^a^, ^b^
Digastric—right (mm)	−0.19 (−0.41 to −0.08)	−0.47 (−1.13 to 0.2)	−0.17 (−0.37 to 0.37)	0.138 (*F* = 2.11)
Digastric—left (mm)	−0.22 (−0.62 to 0.15)	−0.3 (−0.76 to 0)	−0.15 (−0.32 to 0.67)	0.221 (kw = 12.724)

Abbreviations: ES, effortful swallow; *F*, one‐way analysis of variance (post hoc: Tukey test); kw, Kruskal–Wallis variance analysis (post hoc: Mann–Whitney *U* test with Bonferroni correction); MM, Masako maneuver; mV, microvolt.

* *p* < 0.05 statistically significant difference, all descriptive statistics are expressed as median (25th—75th percentile).

Δ difference of pre–post training.

^a^ Significant difference between MM and control group.

^b^ Significant difference between ES and control group.

After 6 weeks of training, the MM group showed improvement in all of the evaluated SM thickness (*p* < 0.05), and the ES group increased mylohyoid (right and left) and anterior belly of digastric muscles (right) thickness (*p* < 0.05) (Table 3). When the post‐training measurements were compared between groups, there was a significant difference in mylohyoid muscle thickness (left) (*p* = 0.002). As a result of pairwise comparisons, ES and MM training groups showed significant improvement in mylohyoid muscle thickness (left) compared with the control group (pairwise comparison: ES–control group *p* = 0.002, MM–control group *p* = 0.015). In addition, both trainings were not superior to each other in terms of any parameter for improving SM thickness (pairwise comparison: ES‐MM group *p* > 0.05) (Table 4).

After 6 weeks of training, both training groups showed improvement in SM strength (ES *p* = 0.0001, MM *p* = 0.014), whereas the control group exhibited no change (*p* = 0.281) (Table 2). When the post‐training measurements were compared between groups, there was a significant difference in SM strength (*p* = 0.002). As a result of pairwise comparisons, ES and MM training groups showed significant improvement in SM strength compared with the control group (pairwise comparison: ES–control group *p* = 0.007, MM–control group *p* = 0.004). In addition, neither training was superior to the other in terms of improving SM strength (pairwise comparison: ES–MM group *p* = 0.944).

## 4 Discussion

The results of this study showed that, both trainings (1) did not change the SM activity during normal swallow, and effortful swallow; (2) increased the strength and thickness of the SM; (3) and these two trainings did not provide superiority to each other in any of the evaluated parameters.

### 4.1 SMs' Activity

There was no previous study that addressed the impact of MM training on SM activity measured during normal and effortful swallow tasks, but there were three previous studies that addressed the impact of ES training [17, 18, 19]. Unlike our study, these previous studies were conducted in geriatrics. The training protocols were similar to those we used in our study but there were several differences such as ES instruction, training frequency and number of ES repetitions. Despite these differences, the results of all studies showed that ES training did not change sEMG activity during both normal swallow and effortful swallow tasks. In this context, the findings of all three studies were compatible with our study [17, 18, 19].

As it is known normal swallow is accepted as a submaximal muscle activity, meaning that the amount of muscle performance required to execute the task is considerably less than the maximal performance that the relevant muscles have the capacity of producing [7, 26]. A previous study reported that sEMG activity measured during normal swallow reflects approximately 42%–53% of maximal SM contraction [29]. In light of this, the muscle activity measured during the normal swallow task does not accurately represent the maximal SM contraction [26]. Given these facts, it is not surprising that SM activity was not altered during the normal swallow task following exercise training.

On the other hand, unlike normal swallow, for the effortful swallow task, the participant was asked to squeeze hard with all his/her muscles while saliva swallow [8]. In other words, the participant is asked to perform a maximal voluntary SM contraction. Although it is known that effortful swallow task causes extra muscle contraction compared to normal swallow, it is unclear how much SM muscle potential and reserve is utilised by participant [8]. Previous studies have shown that participants perform the effortful swallow task with different strategies [18, 19] and that providing feedback while performing the task results in increased muscular activity [11]. Therefore, no change in sEMG activity during the effortful swallow task, suggests that this may be related to the participant's not using the maximal SM muscle reserve in the system [18, 29]. It is important to consider this situation in future studies and record sEMG during tasks that produce maximal SM contraction.

### 4.2 SM Thickness

To the best of our knowledge, there is no previous study evaluating the effect of ES or MM trainings on SM thickness. This study provides the first evidence that ES and MM trainings can be effective in increasing SM thickness.

Previous researches have demonstrated that ES and the MM are effective in terms of selective SM contraction [2, 11], and considering these findings, the improvement in SM thickness in both training groups was an expected result in our study.

As it is known, multiple factors are important for increase skeletal muscle thickness. These are the type, intensity, frequency and duration of the exercise training [30]. Although the optimal exercise dose is still unclear in the dysphagia literature, it has been reported that these factors should be taken into account when preparing an exercise program [2]. Considering that muscle thickness requires greater loading beyond they are routinely exposed to, the results of this study indicate that ES and MM training used in our study protocol meet this criterion [7]. Although there is some speculation about how long it takes to gain muscle thickness as a result of exercise training, the general consensus is that improvements in muscle thickness are achieved within 5–7 weeks of training [30, 31]. Although the 6‐week training duration in our study was sufficient to demonstrate SM thickness, this duration may be relatively short for maximal muscle thickness increase. These findings suggest that more improvements in SM thickness can be achieved by extending the duration of ES and MM training.

### 4.3 SM Strength

In our study, the jaw opening force was measured which reflects the maximal isometric contraction for SM strength. As a result, this evaluation reflects the maximal performance of the SM, which are the primary muscles involved in jaw opening. This explains the improvement in SM strength, although there was no improvement in SM activity recorded during normal swallow and effortful swallow tasks as a result of our study.

Most importantly, in this study, the improvement in SM thickness and SM strength were largely compatible. Unlike muscle thickness, improvement in muscle strength resulted in the training groups superior to the control group. This may be owing to the fact that muscular strength is a variable metric that is affected by a variety of factors including muscle metabolic activity, structural changes in the nervous system, as well as muscle mass. According to the exercise science literature, strength gains are based on neural adaptation in the early stages of training and muscle thickness in the latter stages of training, which leads to strength improvements [30, 31]. Therefore, the muscle thickness obtained in our study is one of the factors causing the increase in muscle strength, but the contribution of neural factors also contributed to the increase in muscle strength and caused this result.

Another point is that as a result of exercise training, muscle strength and thickness gains are not always at the same rate and increases may not be obtained in a linear relationship [30]. Depending on the characteristics of the exercise training, either muscle strength or muscle thickness may increase more.

Lastly, the SM are collectively involved in for jaw opening movement. Therefore, unlike the muscle thickness evaluation, this parameter shows the strength of whole SM, not the individual strength. In this case, the increase in thickness in each muscle is combined and reflected in the muscle strength assessment collectively.

As a result of previous research findings [8] that led us to conduct this study, we expected ES training to be superior to MM training in terms of SM gains. Because this previous study concluded that ES induces more selective SM activity than any other swallow task, including MM.

We think that the unexpected result is related to the situation mentioned above, because each participant performed the effortful swallow task with different strategies. In addition, in the exercise training, the participant may have exhibited less than the maximal effort requested, which may have limited the training effect of ES. Therefore, giving participants feedback is likely to result in maximal performance during ES, resulting in better results in terms of SM gains.

### 4.4 Limitation and Future Directions

One of the most important limitation of our study is the small sample size. Secondly, we did not do progressive loading in our study, each session was the same and included the same number of exercises. This may have negatively affected gains of exercise training. Third, in this study, the strength and thickness of the SM, which are among the main muscles involved in swallowing, were evaluated. However, how much these gains contribute to swallowing safety and efficiency has not been examined. For this reason, the contribution of these trainings to the swallowing function could not be clearly determined with this study. Finally, the fact that this study was conducted on a healthy young population without swallowing problems is not sufficient to confirm the effect of these trainings in dysphagic and geriatric individuals. Investigation of these important points mentioned in future studies will contribute to dysphagia rehabilitation.

## 5 Conclusion

This study contributed to the literature by investigating the effects of ES and MM trainings, which are frequently used in the clinical setting but lack sufficient evidence for their long‐term effects within the scope of dysphagia rehabilitation. One of the important strength of this study is that reliable and objective evaluation methods were employed in the outcome measures. The results of this study showed that ES and MM trainings increased SM strength and thickness in healthy individuals. These results are valuable in terms of forming a basis for future studies in elderly and individuals with dysphagia. Due to the role of the SM in providing swallowing safety and efficiency, the results of this study suggest that trainings may be effective in improving the safety and efficiency of swallowing by developing the SM in dysphagia rehabilitation. However, future studies are needed to prove the therapeutic potential of these swallow‐specific tasks.

## Ethics Statement

All procedures performed in studies involving human participants were in accordance with the ethical standards of the institutional and/or national research committee and with the 1964 Helsinki declaration and its later amendments. The ethical permission was obtained to perform the study from the Aydin Adnan Menderes University Ethics Committee (Protocol number: E‐15189967‐050.02.04‐145385). In order to protect the original idea of the study according to the ‘Publishing and Intellectual Property Rights’, the clinical trial registration number was not received.

## Consent

Informed consent was obtained from all individual participants included in the study.

## Conflicts of Interest

The authors declare no conflicts of interest.

### Peer Review

The peer review history for this article is available at https://www.webofscience.com/api/gateway/wos/peer‐review/10.1111/joor.13862.

## Data Availability

The datasets generated during and/or analysed during the current study are available from the corresponding author on reasonable request.
